# AI-enhanced inverse design of photonic crystal fiber optical modulator using deep reinforcement learning technique

**DOI:** 10.1038/s41598-026-52039-z

**Published:** 2026-06-23

**Authors:** Nada Yazeed M. Dawood, B. M. Younis, Mohamed Farhat O. Hameed, Hamdi A. El-Mikati, S. S. A. Obayya, Nihal F. F. Areed

**Affiliations:** 1 Electronics and Communications Department, Faculty of Engineering, University of Mansoura, Mansoura, 35516 Egypt; 2 Electronics and Communications Department, Misr Higher Institute for Engineering and Technology (MET), Mansoura, Egypt; 3 Nanoelectronics Integrated Systems Center, Nile University, Giza, 12588 Egypt; 4 Centre for Nanotechnology, October Gardens, Zewail City of Science, Technology and Innovation, 6th of October City, 12578 Giza, Egypt; 5 Mathematics and Engineering Physics Department, Faculty of Engineering, University of Mansoura, Mansoura, 35516 Egypt; 6 Centre for Photonics and Smart Materials, Zewail City of Science, Technology and Innovation, October Gardens, 6th of October City, 12578 Giza, Egypt; 7 Department of Electrical Engineering, College of Engineering, Qassim University, Buraydah, 52571 Saudi Arabia

**Keywords:** Inverse design, Deep reinforcement learning, Multi-dimensional optimization, Phase change materials, Optical modulator, Engineering, Mathematics and computing, Optics and photonics

## Abstract

This paper presents a reinforcement learning (RL)-based inverse design framework for photonic device optimization, where a Deep Q-Network (DQN-RL) agent is directly coupled with three-dimensional finite-difference time-domain (3D-FDTD) simulation environment. The proposed approach eliminates the need for pre-collected training data by enabling autonomous, target-driven exploration of a discrete design space. The framework is applied to optimize a silicon-based D-shaped photonic crystal fiber optical modulator (PCF-OM) incorporating a VO₂ phase-change layer, with the objective of minimizing insertion loss (IL). The proposed DQN-RL model demonstrates fast, stable, and consistent convergence behavior. Across multiple independent training runs with different random seeds, all runs converge to the same optimal solution with negligible variation, confirming the robustness of the learned policy. A comprehensive comparative study against particle swarm optimization (PSO), random search, grid search, and Bayesian optimization (BO), performed under identical design space, computational budget, and hardware conditions, demonstrates that the proposed DQN-RL framework achieves the fastest convergence, reaching the optimal solution within only 12 iterations (~ 3 min), compared to 42, 104, 21, and 103 iterations for PSO, random search, grid search, and BO, respectively. reaching an ultra-low IL of 0.935 dB/mm, significantly outperforming the target value of 2 dB/mm. Using the optimized geometry obtained from DQN-RL framework, the PCF-OM exhibits excellent modulation performance, with an extinction ratio exceeding 280 dB/mm, a maximum modulation depth of 99.9%, and broadband operation. Furthermore, fabrication tolerance analysis confirms robust performance, with IL remaining below 1 dB/mm under ± 5% parameter variations. These results demonstrate that DQN-RL provides a fast, robust, and highly effective inverse-design strategy for complex photonic devices.

## 1. Introduction

**Fig. 1:**
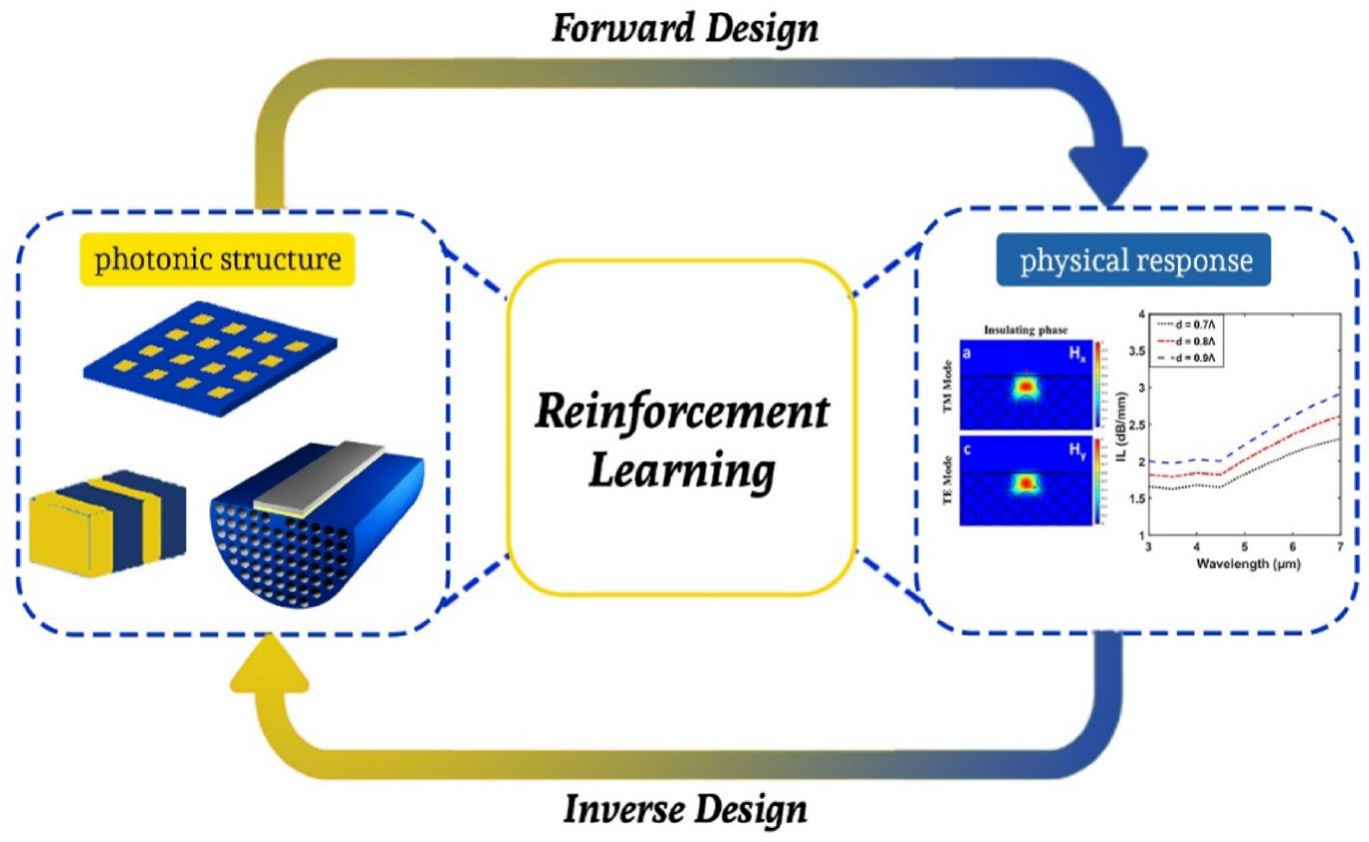
AI paradigms in photonics: The forward modeling approach maps photonic structures to their corresponding optical responses, whereas the inverse design paradigm aims to generate photonic structures that yield a desired optical response.

Inverse design is essential to both the design and development of state-of-the-art optical modulators^1–3^ which have extensive applications in telecommunications^4^, optical interconnects^5^, and photonic integrated circuits^6^. In this context, the inverse problem involves inferring the concealed relationship between an optical response and its associated physical structure, with the objective of retrieving a structural design that produces the desired optical performance, as illustrated in Fig. 1. Since the 1990 s, a wide range of approaches for inverse design in photonics has been introduced, including first-principles methods^7,8^; simulation solvers based on finite element analysis (FEA)^9^ or finite difference time domain (FDTD)^10^, and evolutionary^11,12^ or gradient-based^13,14^ optimization algorithms. Although these traditional methods have historically yielded satisfactory results, they typically require substantial human intervention due to their trial-and-error nature or inherently iterative, which limits their potential for full automation. Since approximately 2012, the emergence of deep learning^15,16^ has offered renewed potential for intelligent inverse design, facilitating the optimization of photonic devices^17,18^. Nevertheless, supervised and semi-supervised learning approaches continue to face three fundamental challenges: (1) the requirement for a substantial, pre-collected dataset for model training, and/or a need for a pretrained multi-layer perceptron (MLP) network integrated within tandem network architectures; (2) the undesired one-to-many mapping problem, which leads to multiple possible design solutions corresponding to the same target optical response; and (3) the incapability of execute further optimization beyond inverse design in photonic structures—meaning that the generated design merely replicates an initial structure without yielding any improvement in its optical performance. Among these issues, the first challenge is particularly difficult to address, as deep learning is well-known for its substantial data requirements. This limitation is further exacerbated by the fact that such datasets are typically generated using computationally expensive physics-based simulations, where a large portion of the sampled data may not contribute effectively to the final optimal design. In contrast, RL provides a fundamentally different paradigm, where data are generated adaptively through interaction with the simulation environment. This enables a goal-directed exploration process, in which only the most relevant samples are collected, thereby improving sample efficiency and reducing redundant simulations compared to conventional data-driven approaches. Furthermore, generating large-scale datasets for photonic device design remains inefficient and often impractical due to the high computational cost of solving Maxwell’s equations, while the limited availability of open-source datasets—owing to the proprietary nature of many designs—further constrains the applicability of supervised learning methods. The second and third challenges also remain significant obstacles for the photonics community. Despite numerous attempts to employ tandem MLP architectures^19^, Variational Autoencoders (VAEs)^20^, Generative Adversarial Networks (GANs)^21^, and iterative deep neural network (DNN)-based optimization methods^22^ for inverse designing of photonic devices, these approaches remain constrained by the aforementioned unresolved limitations. In this context, Reinforcement Learning (RL) was initially proposed as a purely mathematical framework more than fifty years ago^23^, and it has recently demonstrated remarkable success in addressing increasingly complex real-world tasks across various scientific and engineering domains^24–29^. Notable milestones such as Google DeepMind’s AlphaGo Zero^30^ and AlphaStar^31^ exemplify RL’s capacity to reach or even surpass human-level performance in certain tasks. Consequently, RL provides a fundamentally different paradigm that overcomes the challenge of non-uniqueness and enables further optimization of photonic structures beyond inverse design. It offers a distinct advantage in addressing the inverse problem by eliminating the need for pre-collected training data and learning directly through interaction with the environment, where an agent, is trained to select optimal actions in order to maximize the cumulative reward. The effectiveness of this approach relies heavily on the precise formulation of a comprehensive reward function that accurately captures all relevant specifications essential for the desired design outcome^32^. Developing such an effective reward function is critical, as it plays a pivotal role in guiding the agent to produce solutions that are both physically meaningful and technically optimal^33^. Motivated by these advancements, RL holds significant potential to enhance the design and performance of photonic structures, such as photonic crystal fibers (PCFs), with exceptional precision and efficiency—thereby shaping the future direction of photonics research.

In recent years the Deep RL (DRL) that integrates the perceptual capabilities of deep learning with the decision-making framework of RL^34^, has demonstrated remarkable success in solving complex optimization and control problems^35^. By interacting with the simulation environment, DRL agent, typically a neural network (NN), can iteratively learn optimal design policies that efficiently explore high-dimensional design spaces and adapt to nonlinear system responses. This ability makes DRL a promising tool for inverse design in photonics, where adaptive and data-driven optimization is essential.

Against this backdrop of rapid progress in DRL, several studies have begun to explore the application of DRL in photonic design problems. Sajedian et al. conducted two independent studies in which DRL was applied to the optimization of dielectric nanostructures and the design of high-transmission color filters, marking an early milestone in the application of RL to photonics design^36,37^. Building on this progress, Sui et al. successfully employed DRL to perform the inverse design of digital nanomaterials, demonstrating robust convergence of their model^38^. Beyond photonics, Mirhoseini et al. framed the chip floorplanning problem as a DRL task in 2021, leading to the design of Google’s tensor processing unit (TPU) accelerators that outperformed state-of-the-art baseline models^39^. More recently, Kuprikov et al. demonstrated a DRL method for controlling of dissipative soliton generation in a mode-locked fiber laser system, further highlighting the versatility of RL in advanced photonic applications^40^. In 2023^41^, Renjie et al. proposed a DRL approach capable of autonomously performing inverse design and optimizing of two distinct photonic crystal nanobeam cavities without prior knowledge, while yielding unique design solutions. Additionally, Haotian et al^42^. introduced a DRL algorithm for the inverse design of complex slow-light photonic crystal waveguides. Most recently, Mahmoud et al.^43^ applied an RL-based inverse design approach to optimize two photonic devices: a photonic crystal fiber (PCF) for zero-dispersion operation and a broadband polarization-insensitive metamaterial absorber. However, despite these advances, the inverse design of optical modulators using DRL algorithms remains unexplored in the existing literature.

In this paper, we present a novel inverse design framework based on a Deep Q-learning reinforcement learning (DQN-RL) approach for the global optimization of optical modulator performance. the proposed method enables an rl agent to autonomously explore a high-dimensional design space and iteratively learn optimal geometrical parameters that achieve the desired optical response. our results demonstrate that increasing the diversity and volume of training interactions improves convergence speed and training stability, allowing the DQN-RL model to generate robust and efficient design solutions. using the optimized parameters, the framework minimizes insertion loss ( IL) the novelty of this work lies in the integration of a DQN-based RL framework with full-wave FDTD simulations for direct, target-driven optimization of photonic structures in a discrete parameter space, without relying on gradient information or pre-collected datasets. this capability makes the approach particularly suitable for complex photonic structures, where conventional methods often struggle. compared to manual and baseline optimization techniques, the proposed DQN-RL framework provides a clear advantage in terms of convergence speed, stability, and computational efficiency. in addition, the learning-based nature of DQN-RL makes it inherently more scalable and adaptable to higher-dimensional and more complex photonic design problems, where traditional methods may become inefficient or computationally prohibitive. to the best of the authors’ knowledge, this work represents the first implementation of a DQN-RL framework for the inverse design and optimization of optical modulator with a wide range of tunable parameters. the proposed method achieves a globally optimized design solution with superior performance and efficiency. the upcoming sections cover the design and inverse optimization of the pcf optical modulator (sect. 2.1), an overview of reinforcement learning and the proposed DQN-RL-based inverse design strategy (sect. 2.2–2.3), and the implementation of the optimization framework (sect. 2.4). section 3.1–3.4 present and discuss numerical results, demonstrating the effectiveness of the proposed approach. finally, sect. 4 concludes the paper.

## 2. PCF optical modulator and AI-based design methodology

### 2.1. PCF optical modulator (PCF-OM) design and objectives

**Fig. 2:**
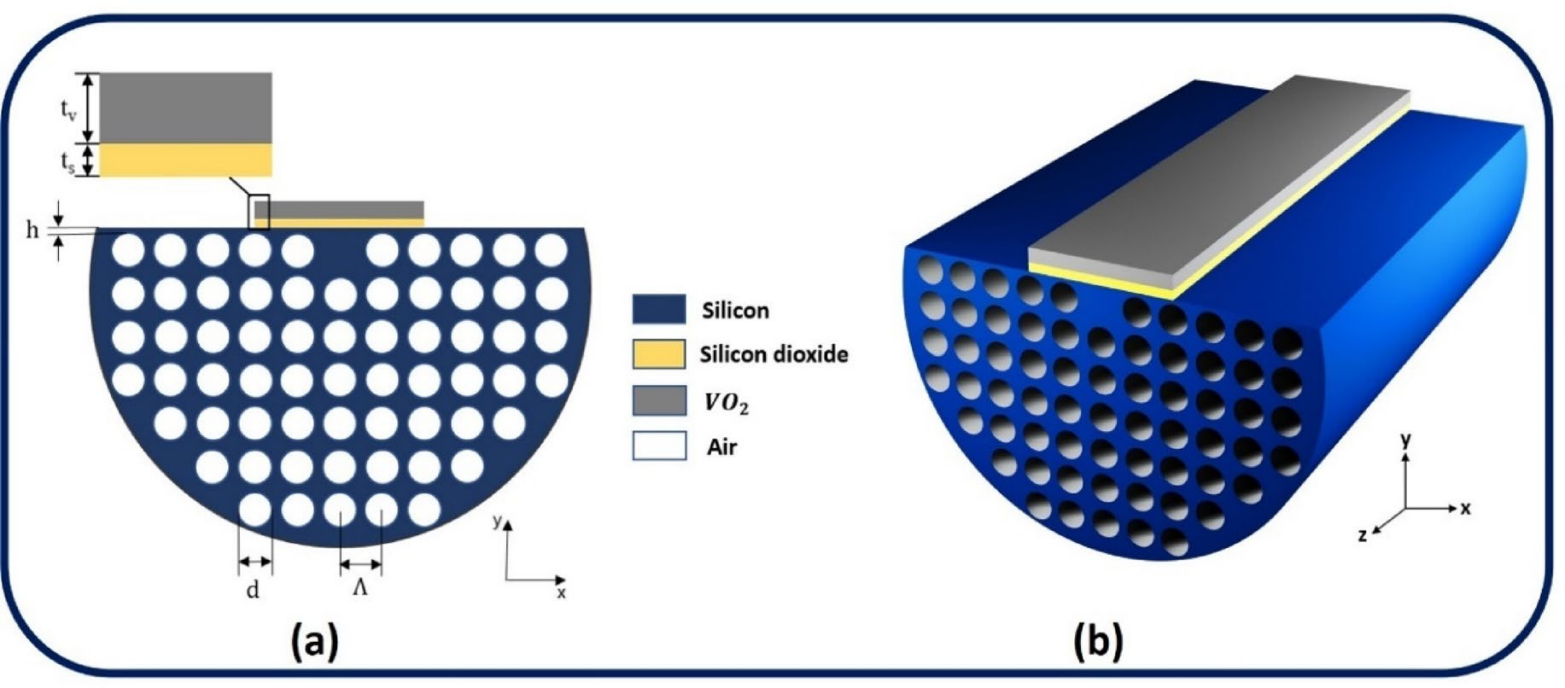
(**a**) 2D and (**b**) 3D schematic of the proposed PCF-OM with a silicon background material coated with a VO_2_ layer (thickness $\:{t}_{v}\:=\:70\:nm$) deposited above SiO_2_ layer as a dielectric spacer (thickness$\:\:{t}_{s}\:=\:20\:nm$). The air hole pitch ($\:\Lambda$) and diameter ($\:d$) are 2.5 μm and 0.92 μm, respectively.

To evaluate the effectiveness of the proposed RL-based inverse design framework, we applied it to improve the performance of an optical modulator reported in Ref^44^. Fig 2a and b present the two-dimensional (2D) and three-dimensional (3D) views, respectively, of the proposed silicon-based D-shaped PCF-OM. The structure features a square-lattice arrangement of air holes embedded within a silicon (Si) background, which was chosen for its excellent transparency in the mid-infrared (mid-IR) region^45^. The air hole diameter is denoted by$\:\:d$, and $\Lambda$ represents the center-to-center spacing (pitch) between adjacent holes. The polished flat surface of the D-shaped fiber is positioned at a vertical distance $\:h$ from the top surface of the air holes. To enable optical modulation, a thin vanadium dioxide (VO₂) layer is integrated near the core region, with a thickness $t_{v}$ and a width of $\:4\Lambda$. VO_2_ is a well-known phase-change material. The phase/state of VO_2_ material can transition from an insulating, optically transmissive monoclinic state to a conducting, optically opaque tetragonal rutile state^46^. This transition can be triggered by various external stimuli, including thermal excitation^47^, electric fields^48^, photoexcitation^49^, doping^50^, and mechanical strain^51^. Placing the VO₂ layer near the fiber core enhances light–material interaction, thereby improving the modulator’s efficiency. A thin silica (SiO₂) layer, serving as a dielectric spacer, is placed beneath the VO₂ layer with a thickness of $t_{s}$ . Due to the metal–insulator transition with a large refractive index contrast^52^, the reported optical modulator operates in two distinct states: the ON and OFF states. In the ON state, VO₂ is in its insulating phase, where the extinction coefficient (κ; imaginary part of VO_2_ refractive index) is very small^52^. Conversely, in the OFF state, VO₂ is in its conducting phase and exhibits a significantly larger κ^52^. The refractive indices of VO₂ in both its insulating and conducting states are taken from^52^. The refractive index dispersion of silicon in the mid-IR region is modeled using the well-known Sellmeier Eq^53^:

1 $$\:{n}^{2}\left(\lambda\:\right)=\epsilon\:+\frac{A}{{\lambda\:}^{2}}+\frac{B{{\lambda\:}_{1}}^{2}}{{\lambda\:}^{2}{{+\lambda\:}_{1}}^{2}}$$

where λ is the wavelength in micrometers, and the constants are: $\lambda _{1}$ = 1.1071$\mu m$ , $\varepsilon$ = 11.6858, $A$ = 0.939816 $\mu m^{2}$ , and $B$ = 8.10461 × 10^−3^. For SiO₂, the refractive index across from 0.21 to 7 μm wavelength range is calculated using the following Sellmeier Eq^54^:

2 $$\:{n}^{2}\left(\lambda\:\right)=1+\frac{0.6961663\:{\lambda\:}^{2}}{{\lambda\:}^{2}-{\left(0.0684043\right)}^{2}\:}+\frac{0.4079426\:{\lambda\:}^{2}}{{\lambda\:}^{2}-{\left(0.1162414\right)}^{2}\:}+\frac{0.8974794\:{\lambda\:}^{2}}{{\lambda\:}^{2}-{\left(9.896161\right)}^{2}\:}$$

The experimental feasibility of the proposed VO₂-embedded D-shaped photonic crystal fiber (PCF) is supported by several established fabrication and integration techniques^55^. D-shaped and side-polished fiber geometries inherently provide a flat and accessible surface, which has been widely utilized for the deposition of metallic layers and external functional materials. Previous studies have demonstrated the successful integration of metallic coatings and electrode structures onto optical fibers and PCFs using standard thin-film deposition techniques^56–58^^59^, confirming the feasibility of applying electrical bias in fiber-based photonic devices. In this context, electrically driven VO₂ switching can be implemented using graphene-assisted electrode configurations, where a VO₂ thin film is combined with a conductive graphene layer connected to metallic electrodes (e.g., Ti/Au). The graphene layer serves as a transparent conductive interface, enabling efficient current injection and uniform thermal distribution required to trigger the VO₂ phase transition^60^. Notably, such electrode-driven VO₂ devices have been experimentally demonstrated at significantly smaller geometric scales than the proposed structure, further supporting the feasibility of electrode integration in the present design. From a fabrication perspective, the proposed structure can be realized using standard thin-film processing techniques. The flat surface of the D-shaped fiber can first be prepared, followed by deposition of the SiO₂ intermediate layer using low-pressure or plasma-enhanced chemical vapor deposition. Subsequently, the VO₂ layer can be deposited via sputtering or pulsed laser deposition, after which a graphene layer is transferred, and metallic electrodes (Ti/Au) are patterned using photolithography and lift-off processes. Upon applying an external voltage, current flows through the graphene layer, inducing Joule heating and/or an electric field that drives the VO₂ phase transition, thereby modulating the optical properties of the guided mode. The electrodes are positioned away from the fiber core to minimize optical perturbation. In addition to electrical actuation, the VO₂ phase transition can also be triggered optically or thermally^61^^62^, providing alternative implementation pathways. These considerations indicate that the proposed design is compatible with current fabrication technologies despite its compact geometry.

The objective of this work is to determine an optimum OM structure that minimizes the IL, which corresponds to the optical confinement loss in the ON state (when VO₂ is in its insulating phase). To achieve efficient modulation performance and surpass previously reported results, the IL should be reduced to below 2 dB/mm. To accomplish this, an inverse design approach is adopted. Traditionally, inverse design in photonics has relied heavily on human intuition and manual parameter tuning, often resulting in time-consuming and suboptimal outcomes. In contrast, RL-driven inverse design framework offers an autonomous and efficient alternative, capable of intelligently exploring high-dimensional design spaces to identify globally optimized solutions without human intervention. In this study, IL is calculated from the imaginary part of the effective refractive index ($\:{n}_{eff}$) using the following equation:

3 $$\:IL\:=\:8.686\:\times\:\:\frac{2\pi\:}{\lambda\:\left(\mu\:m\right)}\:\times\:\:\left\{imag\:\left({n}_{eff}\right)\right\}\times\:\:{10}^{3}\:[dB/mm]$$

where $\:{n}_{eff}$ is the studied mode effective index. All optical responses, including the IL, are computed using full 3D-FDTD simulation. The initial geometrical parameters of the proposed structure are listed in Table 1 and are selected based on wafer constraints, fabrication feasibility, and application-specific requirements.

To address this inverse design problem, a DQN-RL framework is employed to minimize IL and determine the optimal geometrical parameters of the PCF-OM structure.

**Table 1:** Initial geometrical parameters of the suggested structure.

Parameter	$\:{t}_{v}\:\left(nm\right)$	$\:{t}_{s}\:\left(nm\right)$	$\:h\:\left( {nm} \right)$	$\:d\:\left(\mu\:m\right)$	$\:\Lambda \left( {\mu \:m} \right)$
Value	70	20	150	0.92	2.5

### 2.2. Artificial intelligence-based RL

The RL provides an autonomous decision-making framework for inverse photonic design, in which optimal design policies are learned to achieve targeted optical performance objectives^63^. This process is formulated as a Markov Decision Process (MDP), which models the sequential nature of decision-making in a dynamic environment. Within this framework, an autonomous agent interacts with the environment to make decisions that influence both current and future states while maximizing the cumulative reward $\:\left({R}_{t}\right)$, defined as:

4 $$\:{R}_{t}=\sum\:_{t}^{T}{\gamma\:}^{t}{r}_{t}$$

Here, $\:{r}_{t}$ denotes the immediate reward at time step $t$, and $\gamma \in$ [0, 1] represents the discount factor, selected to balance the trade-off between immediate and future rewards. Within this MDP framework, the environment is characterized by a finite set of states $\:{s}_{1}$, $\:{s}_{2}$, $\:{s}_{3}$, …, $\:{s}_{N}$, each corresponding to a distinct configuration of the design problem. The agent selects actions $\:{a}_{1}$, $\:{a}_{2}$, $\:{a}_{3}$, …, $\:{a}_{N}$, to induce transitions between states according to the reward function $\:R\left({s}_{t},\:{a}_{t},\:{s}_{t+1}\right)$, which assigns a scalar value to each state–action–next-state transition. A policy $\pi$ ∶ $\:{s}_{t}$ → $\:{s}_{t+1}$ defines the strategy that maps states to actions, guiding the agent’s behavior. The ultimate goal is to identify the optimal policy $\:{\pi\:}^{*}$ that maximizes the expected cumulative reward over time, enabling efficient exploration and optimization of the photonic design space.

### 2.3. DQN-RL framework design

Generally, Q-learning is an off-policy, model-free RL algorithm that updates value estimates using temporal-difference (TD) learning. In this approach, the agent updates its current estimate by measuring the difference between successive estimations, without requiring an explicit model of the environment. The Q-value $\:Q({s}_{t},\:{a}_{t})$ represents the expected cumulative reward for that specific state–action pair. All Q-values are stored in a table, from which the agent chooses actions according to a policy $\:\pi\:$. The optimal Q-value function, $\:{Q}^{{\pi\:}^{*}}({s}_{t},\:{a}_{t})$, is defined as the maximum expected return achievable by following any policy, and is given by:

5 $$\:{Q}^{{\pi\:}^{*}}({s}_{t},\:{a}_{t})={max\mathbb{E}}_{\pi\:}\left[{R}_{t}\mid\:{s}_{t}=s,{a}_{t}=a\right]$$

During training, the agent gradually learns and converges toward the optimal policy $\:{\pi\:}^{*}$, which yields the maximum expected cumulative reward. The optimal Q-function satisfies an important identity known as the Bellman equation, which expresses the recursive relationship between Q-values of consecutive state–action pairs:

6 $$\:Q({s}_{t},\:{a}_{t})={R}_{t}+\gamma\:\underset{{a}_{t+1}}{max}\:Q\left({s}_{t+1},\:{a}_{t+1}\right)$$

After transitioning from state $\:{s}_{t}$​ to state $\:{s}_{t+1}$​, the Q-value is refined by:

7 $$\:{Q}^{new}({s}_{t},\:{a}_{t})=Q({s}_{t},\:{a}_{t})+\alpha\:\left({R}_{t}+\gamma\:\underset{{a}_{t+1}}{max}\:Q\left({s}_{t+1},\:{a}_{t+1}\right)-Q({s}_{t},\:{a}_{t}\right)$$

where α ∈ [0, 1] is the learning rate. In discrete environments with limited state–action combinations, Q-values can be stored in a lookup table. However, in high-dimensional or continuous design spaces, this approach becomes infeasible due to exponential memory growth and poor generalization. To overcome these limitations, **Deep Q-Network** (DQN) approximate the Q-function using a deep neural network (DNN), which maps state vectors to Q-values for all possible actions. This enables efficient generalization and greatly reduces memory requirements, as the Q-function is represented by a compact set of trainable parameters rather than a large table. Additionally, the DNN enables the agent to learn informative feature representations directly from raw input data, thereby improving learning stability and performance in complex tasks.

In this work, a double-network architecture is employed, consisting of an action network and a target network. The action network $\:Q(s,a,\theta\:)$ predicts Q-values for decision-making, while the target network $\:{Q}^{*}(s,a,\stackrel{-}{\theta\:})$ provides stable target values for learning. By periodically updating the target network, the DQN stabilizes the learning process by preventing oscillations and divergence caused by rapidly shifting target values. The target Q-value is computed as:

8 $$\:{Q}^{*}({s}_{t},\:{a}_{t},\stackrel{-}{\theta\:})={R}_{t}+\gamma\:\underset{{a}_{t+1}}{max}\:{Q}^{*}\left({s}_{t+1},\:{a}_{t+1},\stackrel{-}{\theta\:}\right)$$

where $\:\theta\:$ and $\:\stackrel{-}{\theta\:}$ denote the weights of the action and target networks, respectively.

The network parameters are optimized by minimizing the mean squared error (MSE) between the predicted Q-values from the action network and the target Q-values:

9 $$\:L\left(\theta\:\right)={\left({Q}^{*}({s}_{t},\:{a}_{t},\stackrel{-}{\theta\:})-Q({s}_{t},\:{a}_{t},\theta\:)\right)}^{2}$$

By minimizing loss function $\:L\left(\theta\:\right)$ using gradient descent, the network learns to approximate the optimal Q-values, thereby promoting stable convergence and improved learning efficiency in complex photonic design tasks. It is evident that the neural networks (NNs) form the core of the DQN-based RL inverse design framework. In this work, a fully connected neural network (FCNN) is adopted for both the action and target networks, offering a balance between efficiency and simplicity. An FCNN comprises an input layer, one or more hidden layers, and an output layer, where each neuron in a given layer is connected to all neurons in the preceding layer. These networks are capable of capturing complex patterns and hierarchical features from input data, which allows them to perform various tasks, including classification^64^, regression^65^, pattern recognition^66^, and function approximation^67^. In FCNNs, function approximation is realized through a sequence of layers, each executing two operations: a comprehensive linear transformation $\:{g}_{l,}$, where every output element is influenced by all input elements, and a localized non-linear transformation $\:{\sigma\:}_{l}$. Specifically, for the $\:{l}^{\mathrm{t}\mathrm{h}}$ layer, $\:{g}_{l}:{\mathbb{R}}^{{n}_{l-1}}\to\:{\mathbb{R}}^{{n}_{l}}$ is formulated as $\:{g}_{l}\left(\mathrm{x}\right)={\mathrm{W}}_{l}\mathrm{x}+{\mathrm{b}}_{l},$ where $\:{\mathrm{W}}_{l}\in\:{\mathbb{R}}^{{n}_{l}\times\:{n}_{l-1}}$ and $\:{\mathrm{b}}_{l}\in\:{\mathbb{R}}^{{n}_{l}}$ represent the weight and bias, respectively. The local transformation $\:{\sigma\:}_{l}:{\mathbb{R}}^{{n}_{l}}\to\:{\mathbb{R}}^{{n}_{l}}\:$is applied element-wise as a nonlinear activation function where a layer $\:{\mathcal{L}}_{j}$ of the network is defined as $\:{\mathcal{L}}_{j}\left(\mathrm{x}\right)=\sigma\:\left({\mathrm{g}}_{l}\left(\mathrm{x}\right)\right),$ and thus the entire network with $L$ layers can be expressed as a nested composition of layers:

10 $$\:f\left(\mathrm{x}\right)={\mathcal{L}}_{L}\circ\:{\mathcal{L}}_{L-1}\circ\:\dots\:\circ\:{\mathcal{L}}_{1}\left(\mathrm{x}\right)$$

where, the set of all network parameters $\:\left\{{\mathrm{W}}_{1},{\mathrm{b}}_{1},{\mathrm{W}}_{2},{\mathrm{b}}_{2},\dots\:,{\mathrm{b}}_{l}\right\}$ is collectively denoted as $\theta$. The process of refining the parameters $\theta$ of the network to meet problem-specific requisites, thereby yielding a solution, is known as training.

In general, training entails iteratively updating the network parameters to minimize a chosen loss (or cost) function, which measures the difference between the predicted outputs and the actual target values. Let $\:\mathrm{X}=\left({\mathrm{x}}_{1},{\mathrm{y}}_{1}\right),\left({\mathrm{x}}_{2},{\mathrm{y}}_{2}\right),\dots\:,\left({\mathrm{x}}_{N},{\mathrm{y}}_{N}\right)$ represent a set of $N$ training samples, where each pair $\:\left({\mathrm{x}}_{i}\in\:{\mathbb{R}}^{n},{\mathrm{y}}_{i}\in\:{\mathbb{R}}^{m}\right)$ corresponds to an input feature vector and its associated target output. The goal is to find the optimal parameter set $\theta$ that minimizes the empirical loss, expressed as:

11 $$\:\underset{\theta\:}{min}\:\frac{1}{N}\sum\:_{i=1}^{N}\:l\left({\mathbf{y}}_{i},f\left({\mathbf{x}}_{i};\theta\:\right)\right)$$

In this context, $l$ denotes the chosen loss function, such as mean squared error or cross-entropy loss. The optimization problem is commonly solved using gradient-based methods, where the gradient $\:{\nabla\:}_{\theta\:}l(\mathbf{y}$,$\:\:f(\boldsymbol{x};\theta\:))$ of the loss function with respect to the network parameters is computed through backpropagation and used to iteratively reduce the loss. This analytical formulation of the FCNN was described in^68^. In our work, a four-layer FCNN is implemented, consisting of an input layer representing the state vector, two hidden layers with 80 and 120 neurons, respectively, and an output layer producing Q-values for all possible actions. This structure enables the agent to select the optimal action based on the estimated Q-values.

### 2.4. DQN-RL framework for inverse design

In this study, The DQN-RL framework is defined by six principal components: state space, action space, environment, reward function, target network, and memory buffer.

- **State:** The state represents the current configuration of the PCF-OM, characterized by its key geometrical parameters: VO₂ thickness ($\:{t}_{v}$), SiO₂ thickness ($\:{t}_{s}$), distance from the air holes to the top edge $\:\left(h\right)$, air-hole diameter ($\:d$), and pitch between adjacent air holes ($\:\Lambda$).
- **Action:** Each action corresponds to a discrete modification of the PCF-OM geometrical parameters. Once selected, the action is applied directly to the FDTD environment, which reconstructs the updated PCF-OM geometry and computes the resulting optical response, generating the next state and reward for the learning algorithm. The action is expressed as an $m \times 1$ vector, where $m$denotes the number of design geometrical parameters. Each element in this vector takes one of three discrete values: 0, 1, or 2 indicating a decrease, no change, or an increase in the corresponding parameter, respectively (e.g., an action such as [0 2 1 2 0]). Accordingly, the output layer of the NN comprises $\:{3}^{m}$ neurons, enabling systematic exploration of the design space while maintaining physical constraints. Here, with $\:m=5$, the total number of possible discrete action combinations is $\:{3}^{5}=243$, representing all feasible design perturbations.
- **Environment:** The environment refers to a FDTD-based simulation implemented in Lumerical software package^69^, which solves Maxwell’s equations to evaluate optical performance of the structure. Upon receiving an action from the agent, the environment updates the structural geometry, computes the resulting optical responses, and returns the next state and corresponding reward. Since full FDTD simulations are computationally intensive, a DNN may optionally be used to approximate the environment’s response, reducing simulation time and computational cost.
- **Reward:** The reward quantifies how closely the current design meets the desired performance target. In this study, the objective is to minimize the IL below a target threshold of $\:{IL}_{target}=2\:dB/mm\:$for efficient modulation. Given a selected action $\:{a}_{t}$ under state $\:{s}_{t}$, the reward is computed as:

12 $$\:{r}_{t}\:=\:(100\:-\:(IL-\:{IL}_{target})\:\times\:\:50)$$

This formulation is designed to provide a target-driven optimization signal, where the agent is explicitly guided toward achieving a predefined target threshold of $\:{IL}_{target}=2\:dB/mm$. Unlike generic reward formulations that only minimize a quantity, this approach encourages convergence toward a physically meaningful performance threshold, which is critical for practical optical modulator design. The linear structure of the reward function is intentionally chosen to ensure smooth and stable learning behavior. By maintaining a continuous and monotonic relationship between IL and the reward, the agent can clearly distinguish between better and worse design states, which improves convergence stability and avoids abrupt reward fluctuations. Furthermore, the scaling factors in Eq. (12) are carefully selected to enhance training efficiency. The constant term (100) ensures that the reward remains within a numerically stable and predominantly positive range during training, while the scaling coefficient (50) amplifies the sensitivity of the reward to variations in IL. This allows the agent to more effectively differentiate between candidate designs, particularly in the vicinity of the target IL, thereby accelerating convergence. It is also important to note that the chosen reward formulation reflects a balance between optimization sensitivity and training stability. While alternative nonlinear or multi-objective reward formulations could be considered, the adopted linear form was found to provide robust convergence behavior and consistent performance improvements in our problem setting.

- **Target network:** A separate target network is employed, with its weights ($\:\stackrel{-}{\theta\:}$) periodically updated from the action network. This “freezing” mechanism reduces correlations between predicted and target Q-values, thereby mitigating oscillations and enhancing the convergence stability of the learned policy.
- **Memory buffer:** The DQN framework incorporates a memory buffer in which the agent stores its interactions with the environment as tuples ($\:{s}_{t},\:{a}_{t}$, $\:{R}_{t}$, $\:{s}_{t+1}$). During training, a mini-batch is randomly sampled from this buffer to update the neural-network weights. This mechanism improves sample efficiency by reusing past experiences, reduces temporal correlation through random sampling, and enhances training stability by smoothing updates and preventing divergence.

**Fig. 3:**
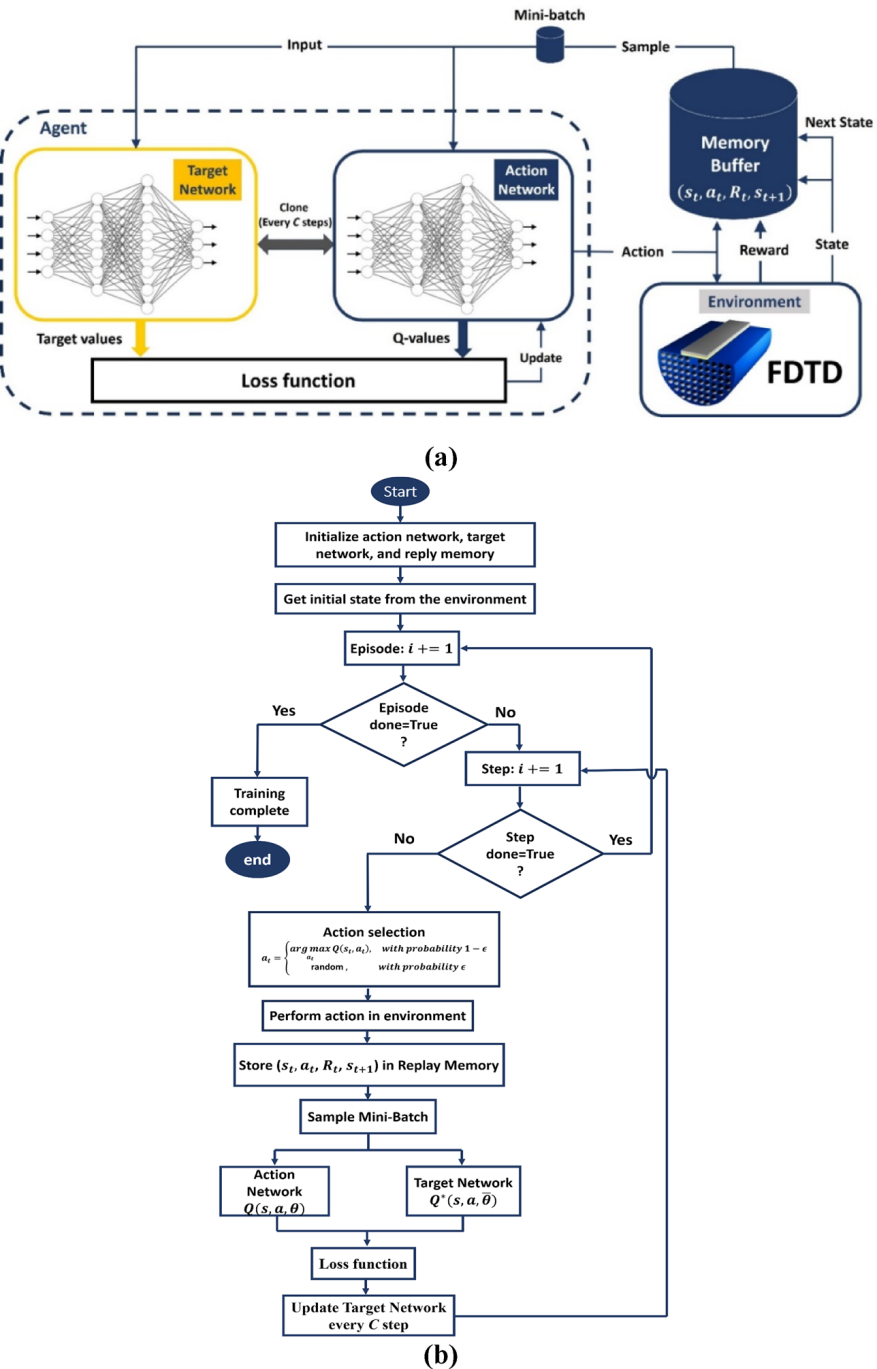
(**a**)Inverse design approach utilizing DQN-RL for optimizing a PCF-OM simulated through Lumerical 3D-FDTD, and (**b**) Flow chart diagram.

Figure 3 shows the end-to-end workflow and the flow chart diagram of the proposed DQN-RL inverse-design framework for optimizing the PCF-OM structure. Once the problem components are defined, the learning process proceeds through a closed feedback loop driven by a high-fidelity 3D-FDTD simulation environment. After the agent selects an action, the corresponding geometric update is automatically applied to the PCF-OM model, and the FDTD solver performs a full modal analysis—executing 1000 iterations to ensure stable electromagnetic field convergence and reliable IL extraction. The resulting optical response defines the next state and provides the reward that guides the agent’s learning. Within this loop, the action network estimates the Q-values associated with the current state and is continuously refined through gradient-based updates. In contrast, the target network is held fixed and refreshed only every *C* iterations to synchronized with the action network, a strategy that provides a stable reference during TD updates and suppresses oscillations. The predicted and target Q-values are then used to compute the loss function, as shown in Eq. 9, which is minimized using gradient-based optimization via the RMSprop algorithm with a learning rate of 0.00025. To prevent premature convergence to suboptimal policies, an epsilon (ϵ)-greedy policy is adopted to balance exploration and exploitation. With probability (1-ϵ), the agent selects the action with the highest Q-value (exploitation), while with probability ϵ, it selects a random action (exploration):

13 $$\:a_{t} = \left\{ {\begin{array}{*{20}c} {\mathop {\arg \max }\limits_{{a_{t} }} Q\left( {s_{t} ,a_{t} } \right),\:\:\:\:with\:probability\:1 - \in } \\ {\:random,\:\:\:\:\:\:\:\:\:\:\:\:\:\:\:\:\:\:with\:probability\: \in } \\ \end{array} } \right.$$

Here, $\in$ is linearly decreased from 0.99 to 0.01, allowing a smooth transition from wide exploration in early stages to fine-grained exploitation as the agent becomes more knowledgeable about the design space. After executing each action, the tuple ($\:{s}_{t},\:{a}_{t}$, $\:{R}_{t}$, $\:{s}_{t+1}$) is stored in the memory buffer, from which random mini-batch is drawn to update the network parameters. The entire inverse-design framework was implemented in Python using the Spyder IDE and developed with the Gym, PyTorch, and NumPy libraires. The simulation and training hyperparameters adopted in this work are summarized in Table 2. Through repeated cycles of action selection, FDTD-based evaluation, and reward-driven updates, the agent incrementally improves its policy, ultimately converging toward a PCF-OM geometry that significantly reduces IL and enhances overall device performance.

**Table 2:** Hyperparameters of the proposed DQN-RL model.

Hyperparameter	Value
Seed ^a*^	42
Mini-batch size for training	64
Discount factor for future rewards ($\:\gamma\:$)	0.99
Initial epsilon for ε-greedy policy	*0.99*
Final epsilon for ε-greedy policy	*0.01*
Epsilon decay rate ^b*^	200
Target network update frequency (*c*)	5
Maximum size of the replay buffer ($\:D$)	*5000*
Number of hidden layers	2
Number of neurons in the first hidden layer	80
Number of neurons in the second hidden layer	120
Number of output neurons (for 3⁵ = 243 discrete actions)	243
Optimizer	RMSprop
Learning rate of the optimizer ^c*^	0.00025
Smoothing constant of the optimizer^c*^	0.95
Momentum of the optimizer^e*^	0.95
Total number of episodes for training	10
Maximum steps per episode	100

^a*^ Seed: A random seed defines the initial state of the pseudo-random number generator, enabling controlled stochastic behavior and reproducible training outcomes in RL experiments.^b*^ Epsilon decay rate: Controls the rate at which the exploration probability (ε) decreases during training. A value of 200 ensures a gradual transition from exploration to exploitation over episodes.^c*^ Learning rate of the optimizer (0.00025): Determines the step size used to update the network weights during training. A small value is selected to ensure stable convergence.^d*^ Smoothing constant of the optimizer (0.95): Corresponds to the decay factor used in the moving average of squared gradients (e.g., in RMSProp), helping to stabilize updates.^e*^ Momentum of the optimizer (0.95): Controls the contribution of past gradients in the current update, accelerating convergence and reducing oscillations.

## 3. Results and discussion

**Fig. 4:**
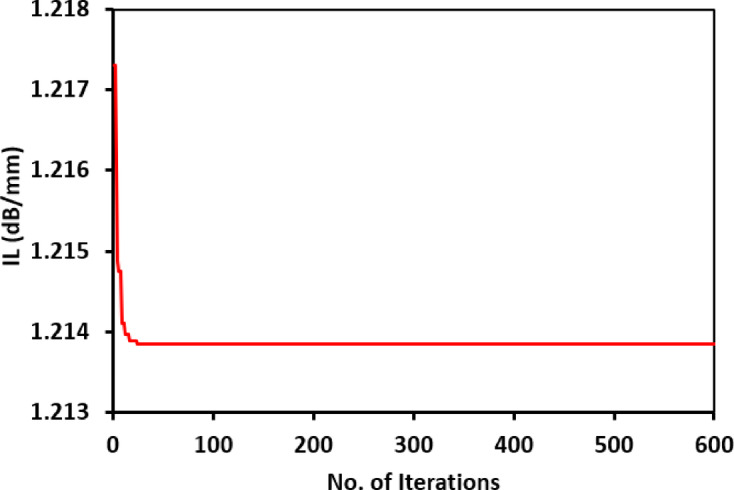
Convergence study of IL with respect to number of iteration where $\:\varDelta\:x$ *and* $\:\varDelta\:y$ are simultaneously updated at each iteration.

To ensure the numerical accuracy of the FDTD simulations, a mesh convergence study was performed prior to the optimization process using the initial geometrical parameters. An automated refinement procedure based on the built-in particle swarm optimization (PSO) tool in Lumerical was employed to vary the mesh sizes ($\:\varDelta\:x$ and $\:\varDelta\:y$) simultaneously within the range of 0.004 to 0.1. The IL was monitored for each mesh configuration, and convergence was identified when further refinement resulted in negligible variation in the IL. As shown in Fig. 4, the simulation results become stable beyond a mesh resolution of $\:\varDelta\:x$ = 0.01 and $\:\varDelta\:y$ = 0.03, indicating numerical convergence after approximately 27 iterations. It was also observed that decreasing the mesh size significantly increases the simulation time due to the higher computational cost associated with finer discretization, highlighting the trade-off between numerical accuracy and computational efficiency. Therefore, these mesh settings were selected as an optimal balance between accuracy and simulation time and were adopted in all subsequent simulations. In addition, perfectly matched layer (PML) boundary conditions were employed to effectively absorb outgoing electromagnetic waves and eliminate artificial reflections, making them particularly suitable for open-region photonic structures such as the proposed PCF-based modulator.

### 3.1. Performance of training DQN-RL model

**Fig. 5:**
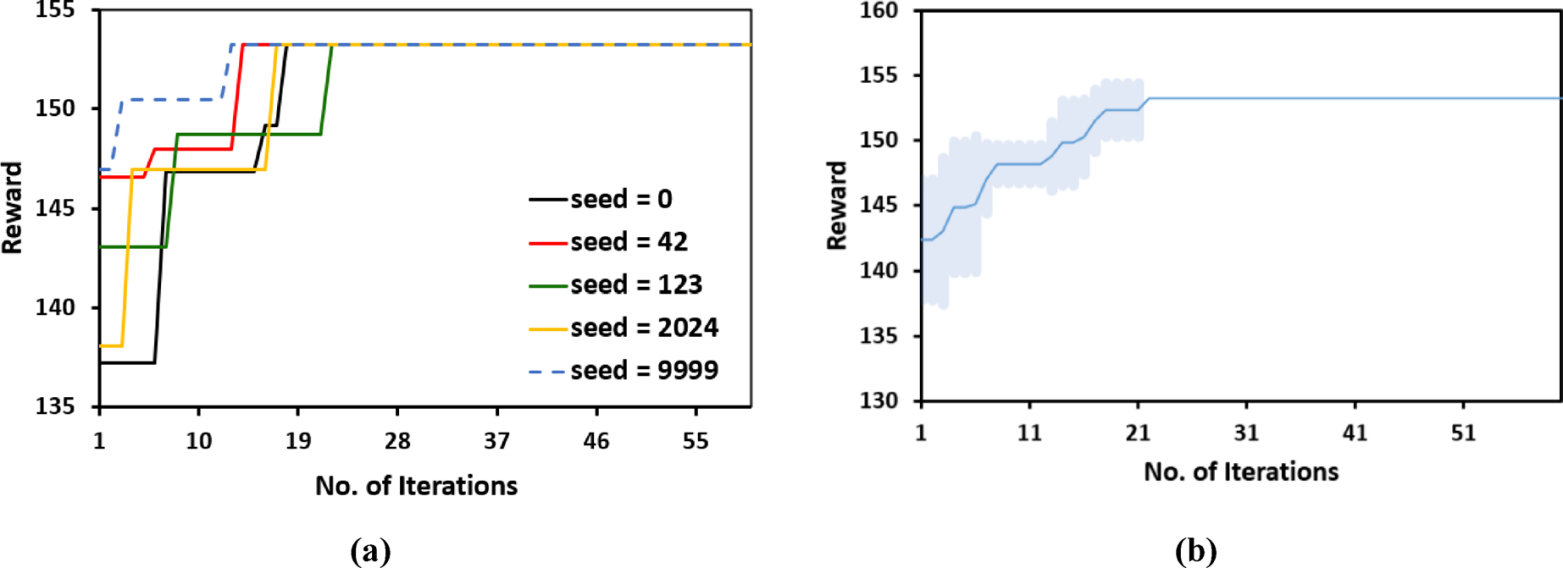
(**a**) Convergence behavior of the DQN-RL model for different random seeds. (**b**) Mean reward and standard deviation, demonstrating consistent convergence and robustness. For clarity, only the initial training iterations are displayed, while the remaining iterations exhibit stable and identical performance.

Figure 5a presents the convergence behavior of the proposed DQN-RL framework evaluated over multiple independent training runs using different random seeds. As shown, all runs exhibit a rapid increase in reward during the early stages of training, followed by stable convergence toward the same optimal solution. Despite minor variations in the initial exploration phase, all seeds consistently reach the maximum reward within a limited number of iterations, demonstrating the robustness of the proposed approach against random initialization. It should be noted that, although all training runs were performed for a total of 1000 iterations, only the initial portion of the convergence curves is shown in the figures for clarity and visualization purposes. This is because the reward values remain stable and nearly identical across all runs after convergence and displaying the full range would obscure the differences observed during the early training stages. Based on the maximum reward obtained during training, the optimized set of geometrical design parameters that meet the target specifications is summarized in Table 3. 

**Table 3:** Optimized geometric parameters of the proposed DQN-RL framework.

Parameter	$\:{t}_{v}\:\left(nm\right)$	$\:{t}_{s}\:\left(nm\right)$	$\:h\:\left(nm\right)$	$\:d\:\left(\mu\:m\right)$	$\:\:\:\Lambda \left( {\mu \:m} \right)$
Value	60	10	200	1.76	2.7

To further quantify this behavior, the mean reward and corresponding standard deviation across all runs are illustrated in Fig. 5b. The narrow spread of the shaded region indicates low variability among different runs, while its gradual disappearance after approximately 20 iterations confirms that all runs converge to the same optimal solution with negligible deviation. This behavior highlights the stability and consistency of the learned policy, as the agent reliably identifies the same optimal design regardless of initialization conditions. Such consistency can be attributed to both the structured nature of the discrete search space and the ability of the DQN agent to efficiently learn and exploit optimal actions through interaction with the simulation environment.

### 3.1.1. Comparative analysis with baseline optimization methods

To rigorously evaluate the performance of the proposed DQN-RL framework, a comprehensive comparative study was conducted against several baseline optimization methods, including particle swarm optimization (PSO), random search, grid search, and Bayesian optimization (BO). To ensure a fair and consistent comparison, all optimization methods were conducted within an identical and explicitly defined design space, where the geometrical parameters were constrained within the same bounds as summarized in Table 4.

**Table 4:** Design parameter bounds used for all optimization methods.

Parameter	Min	Max
$t_{v} (nm)$	60	60
$t_{s} (nm)$	10	30
$\:h$(nm)	100	200
$\:d$(µm)	1.76	1.92
$\Lambda$(µm)	2.3	2.7

It is worth noting that, the full optimization process of DQN-RL is completed within approximately 4 h for 1000 iterations. Additionally, all methods were executed on the same hardware platform (HP ZBook, Intel^®^ Core™ i7-4810 M 2.80 GHz, 16.0 GB DDR3 RAM, 512 GB SSD, NVIDIA Quadro K3100M (4 GB)) and under identical FDTD simulation settings. Furthermore, the parameter space was discretized into three levels per variable, resulting in a finite search space of 3⁵ = 243 configurations, ensuring that all methods operate over exactly the same set of candidate solutions. To maintain full consistency, the PSO algorithm was implemented in Python using a discrete formulation rather than its standard continuous version, allowing it to operate under the same parameter resolution, objective function, IL target, reward formulation, and convergence criteria as the DQN-RL framework. The PSO hyperparameters were selected to ensure stable convergence in this discrete setting, Table 5.

**Table 5:** PSO hyperparameters used in the optimization process.

Hyperparameter	Symbol	Value
Swarm size	N	10
Maximum generations	—	100
Number of dimensions (design parameters)	—	5
Inertia weight	w	0.5
Cognitive coefficient	c_1_	1.5
Social coefficient	c_2_	1.5

**Fig. 6:**
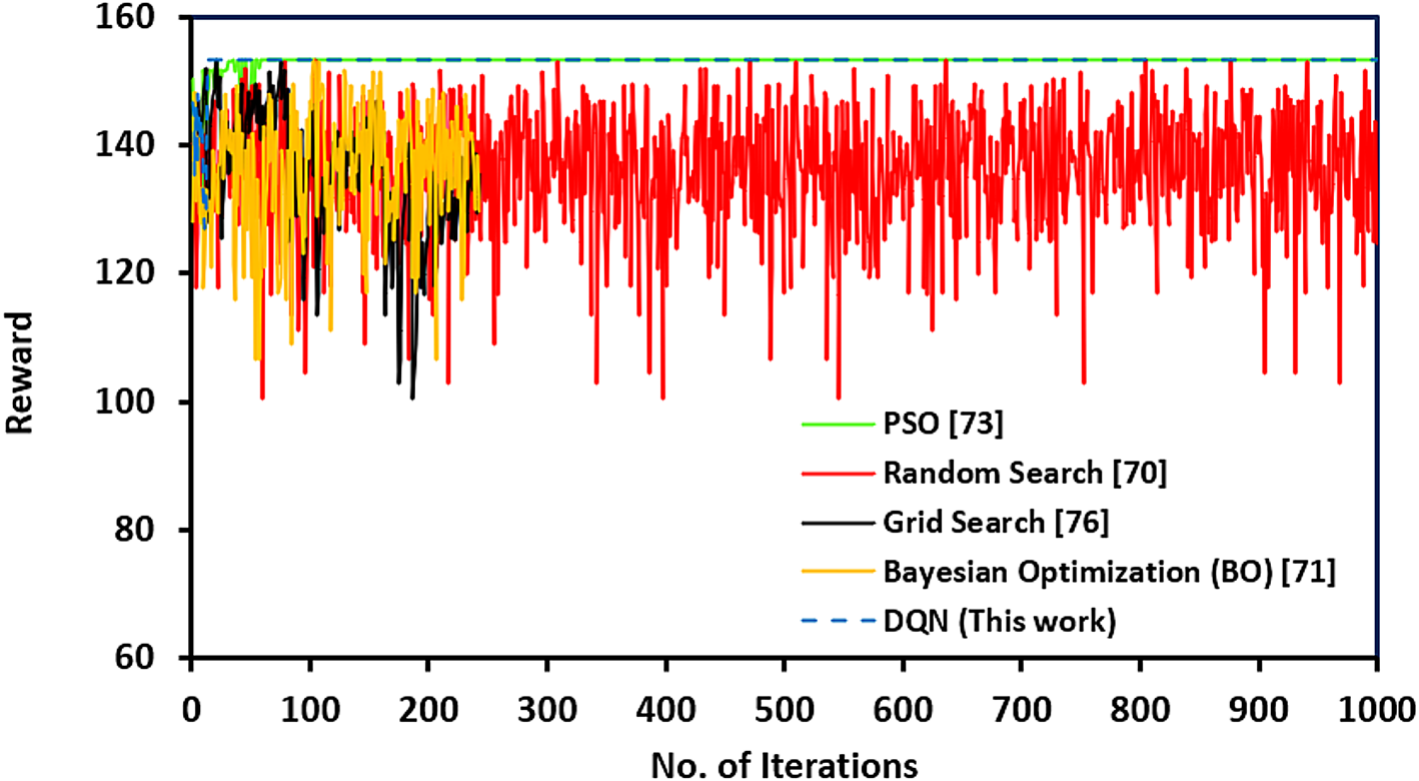
Reward versus iteration for DQN-RL and baseline optimization methods (random search [77], grid search [78], Bayesian optimization [79], and PSO [76]), showing faster and more stable convergence of DQN-RL.

The quantitative results are summarized in Table 6, while the convergence behaviors are presented in Fig. 6. Although the design space is relatively limited, the proposed DQN-RL framework demonstrates the fastest convergence, reaching the optimal solution within only 12 iterations (≈ 3 min), compared to 42 iterations (≈ 13 min) for PSO, 104 iterations (≈ 28 min) for random search, 21 iterations (≈ 6 min) for grid search, and 103 iterations (≈ 33 min) for BO. Among the baseline methods, random search exhibits significant fluctuations due to its purely stochastic nature and lack of guidance toward promising regions of the search space^70^. Grid search, while deterministic, follows a non-adaptive exhaustive strategy that evaluates all possible configurations without prioritization, resulting in inefficient use of computational resources^71^. BO, in contrast, employs a more guided search strategy through surrogate modeling; however, it requires constructing and updating a Gaussian process model at each iteration using all previously observed data. This process involves repeated retraining and matrix inversions, leading to computational complexity that scales cubically with the number of samples^72^. As a result, BO becomes increasingly inefficient as the number of iterations grows, particularly in discrete or moderately sized design spaces such as the one considered in this work.

As illustrated in Fig. 6, the proposed DQN-RL framework not only achieves faster convergence but also exhibits more stable optimization behavior. Although PSO eventually reaches a similar final performance, it requires a significantly larger number of iterations (up to approximately 60 iterations to stabilize near the optimum). The similarity in final IL values between DQN-RL and PSO is attributed to the finite size of the discrete search space; once the optimal configuration is identified, both methods converge to the same solution. However, this comparable final performance does not imply equivalent efficiency. PSO performance typically degrades as the dimensionality of the design space increases, requiring larger swarm sizes and more iterations to maintain effective exploration, thereby increasing computational cost^73,74^. Additionally, PSO may suffer from premature convergence to local optima in highly non-convex or discrete problems^75^and lacks a mechanism for knowledge transfer across runs. In contrast, the proposed DQN-RL framework learns an optimization policy that improves over time, enabling more efficient exploration and enhanced scalability. These results confirm that, even within a relatively small design space, DQN-RL provides superior convergence speed, stability, and computational efficiency, while offering strong potential for extension to more complex and higher-dimensional photonic design problems.

**Table 6:** Performance comparison of DQN-RL and baseline optimization.

Method	Number of iterations to reach optimum	Time to reach optimum (min)	IL (dB/mm)	Reward
PSO [73]	42	13	0.935	153.27
Random search	104	28		
Grid search^76^	21	6		
Bayesian optimization (BO) [71]	103	33		
DQN (this work)	12	3		

### 3.1.2. Performance evaluation of the optimized PCF-OM design

To further evaluate the effectiveness of the optimized PCF-OM, key performance metrics, including ER, MD, and optical bandwidth, were analyzed across the wavelength range of 3–7 μm. These metrics were computed using the geometrical parameters obtained by the DQN-RL framework. The ER is defined as the difference in IL between the conducting (OFF) and insulating (ON) states of the VO₂ layer; ER(λ)=IL_OFF_ (λ) - IL_ON_ (λ)

**Fig. 7:**
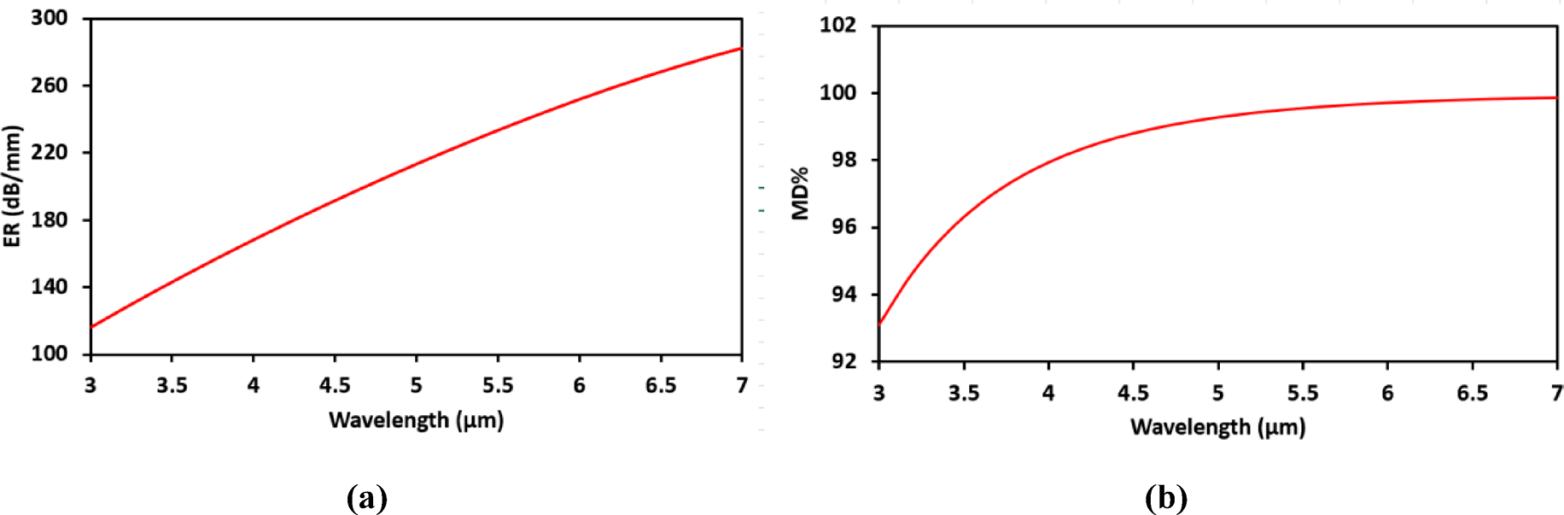
(**a**) ER and (**b**) MD spectrum of the optimized PCF-OM over 3–7 µm wavelength range.

Figure 7a shows the ER spectrum, where the optimized structure achieves exceptionally high ER values exceeding 280 dB/mm near 7.0 μm, indicating strong suppression of the guided mode in the conducting state. Across the entire wavelength band, ER remains consistently high (approximately 120–280 dB/mm), confirming robust switching performance.

The modulation depth (MD) quantifies the relative change in transmission between the conducting (OFF) and insulating (ON) states^77^:

14 $$\:\mathrm{M}\mathrm{D}\left(\lambda\:\right)=\frac{{T}_{\mathrm{O}\mathrm{F}\mathrm{F}}\left(\lambda\:\right)-{T}_{\mathrm{O}\mathrm{N}}\left(\lambda\:\right)}{{T}_{\mathrm{O}\mathrm{F}\mathrm{F}}\left(\lambda\:\right)}\times\:100\mathrm{\%}$$

Since optical transmission is related to IL through $\:T={10}^{-\mathrm{I}\mathrm{L}/10}$, this can be expressed directly from ER as:

15 $$\:\mathrm{M}\mathrm{D}\left(\lambda\:\right)=\left(1-{10}^{-\mathrm{E}\mathrm{R}\left(\lambda\:\right)/10}\right)\times\:100\mathrm{\%}$$

As shown in Fig. 7b, MD attains 99.9% and remains above 93% over the studied spectrum. These high MD values clearly demonstrate robust and uniform switching performance.

### 3.2. Field distribution and light propagation of PCF-OM

**Fig. 8:**
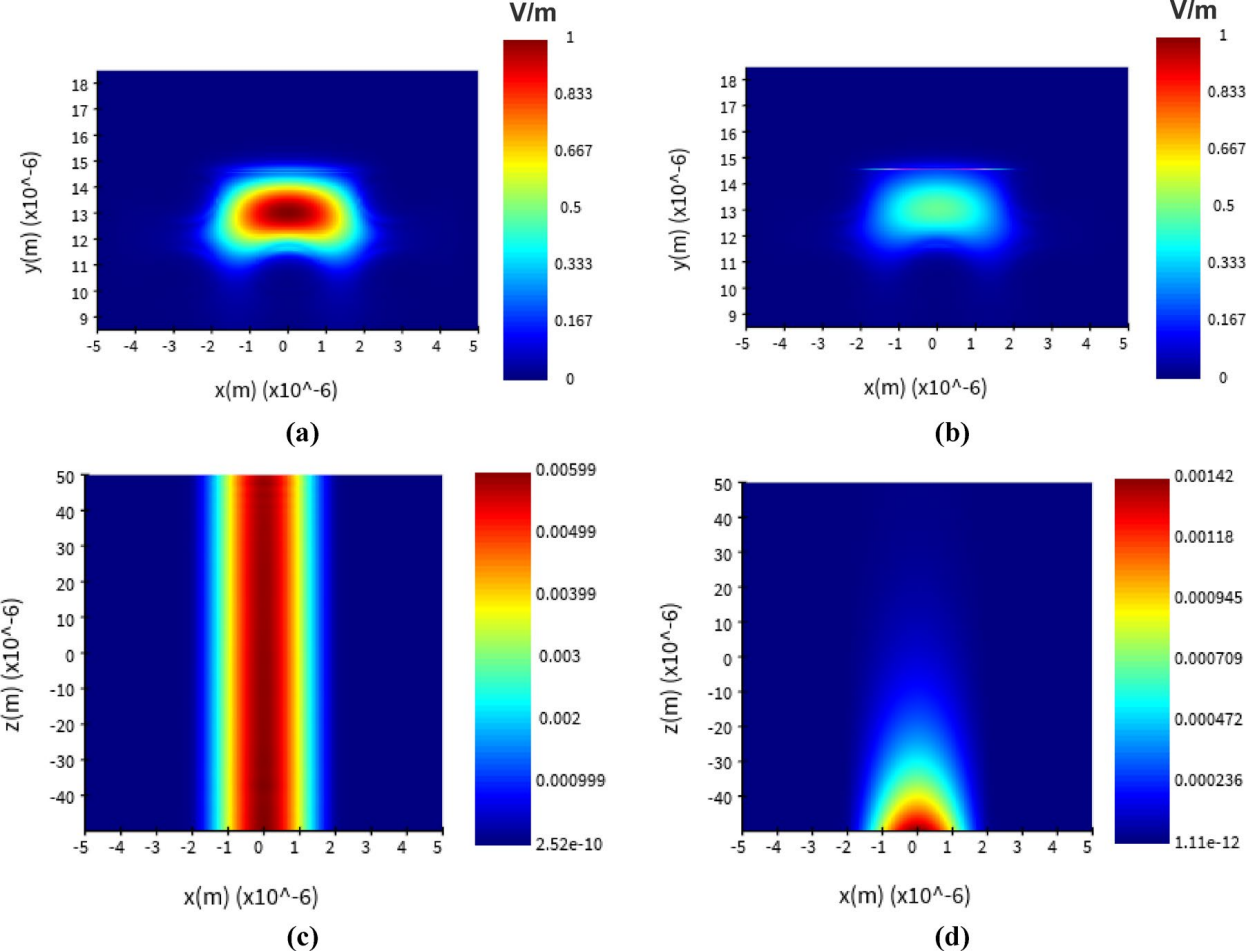
Field distributions of the TM mode in (**a**) the insulating phase (ON state) and (**b**) the conducting phase (OFF state), along with the corresponding light propagation for (**c**) the insulating phase (ON state) and (**d**) the conducting phase (OFF state). All field distributions and propagation patterns are evaluated at a wavelength of $\:\lambda\:=5\:\mu\:m$.

To verify the modulation behavior of the proposed PCF-OM, the electromagnetic field distribution and light propagation were analyzed using the optimized geometrical parameters obtained by the DQN-RL framework, listed in Table 3, at a wavelength of 5 μm. Figure 8 illustrates the field distributions of the fundamental transverse-magnetic (TM) for the two VO₂ states; insulating and conducting. It is worth noting that the TM mode is investigated due to the placement of the VO₂ layer above the core in the vertical direction, where the dominant TM component (Ey) is vertically oriented. This configuration results in a strong interaction between the TM mode and the VO₂ layer, making the TM mode highly sensitive to VO₂-induced modulation. In Fig. 8a, where VO₂ is in its insulating phase, the fundamental TM mode is well confined within the PCF core, exhibiting a very low optical loss. In contrast, when VO₂ transitions to its conducting phase, as shown in Fig. 8b, a significant portion of the optical field is attracted toward the lossy VO₂ layer due to its plasmonic-like absorption characteristics, resulting in pronounced attenuation. Figure 8c and d depict the 3D field propagation along the device’s longitudinal (z) axis over a length of 100 μm. In the insulating phase, Fig. 8c, the TM mode propagates efficiently with negligible absorption. However, in the conducting phase, Fig. 8d, the field exhibits severe attenuation as the TM mode is absorbed by the VO₂ layer, confirming the strong modulation efficiency. Notably, Fig. 8d also demonstrates that the modulation behavior persists even when the device length is reduced below 100 μm, indicating that effective optical switching can be achieved within a shorter device footprint. This miniaturization contributes to reduced fabrication complexity, lower power consumption, and improved integration with compact photonic circuits. Overall, these results confirm that the proposed VO₂-integrated PCF-OM provides efficient modulation of the guided light through the reversible switching of VO₂ between its insulating and conducting states, enabling dynamic and high-contrast control of optical transmission.

### 3.3. Discussion on fabrication uncertainties/tolerances

**Fig. 9:**
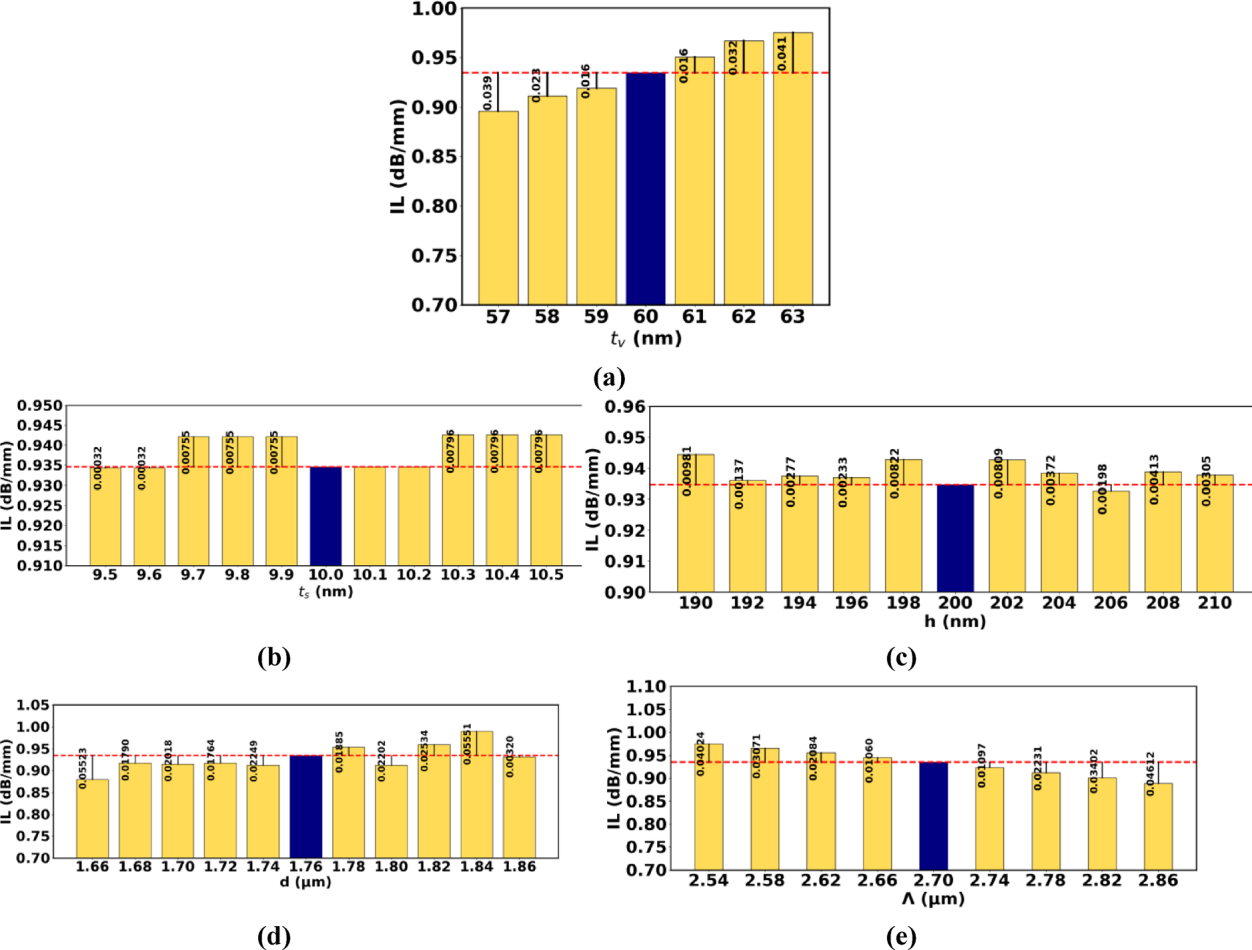
comprehensive fabrication tolerance study for five key geometrical parameters (**a**) $\:{t}_{v}$, (**b**) $\:{t}_{s}$, (**c**) $\:h$, (**d**) $\:d$, and (**e**) $\Lambda$, where each parameter was independently varied by ± 5% of its optimum value while keeping the others fixed at their optimized values.

To evaluate the robustness of the proposed PCF-OM design against fabrication imperfections, a comprehensive fabrication tolerance analysis was performed for five key geometrical parameters: $\:{t}_{v}$, $\:{t}_{s}$, $\:h$, $\:d$, and $\Lambda$. Each parameter was varied by ± 5% of its optimum value independently while keeping all other parameters fixed. As shown in Fig. 9a–e, In each figure, the red dashed horizontal line indicates the optimal IL value corresponding to the nominal design configuration obtained from the DQN-RL optimization, while the blue bar highlights this optimal configuration. The yellow bars represent the IL values obtained resulting from upward or downward deviations (± 5%) of a single parameter, thereby emulating realistic fabrication imperfections. To quantitatively capture the IL sensitivity, the numerical label above each yellow bar indicates the absolute difference between corresponding IL value and the optimal IL. This labeling provides a clear visual comparison of the design’s robustness. For instance, in Fig. 9a, adjusting the VO₂ thickness within ± 3 nm of its optimal value (60 nm) leads to only a modest increase in IL, with the maximum deviation reaching approximately 0.041 dB/mm at 63 nm. Such minimal variation indicates that the device experiences only minor performance degradation under typical thickness fluctuations. Figure 9b–e show similar behavior for the remaining parameters. Across all geometric variations, the IL consistently remains below 1 dB/mm, demonstrating stable optical performance within the ± 5% tolerance window. Therefore, the tolerance analysis confirms that the proposed DQN-RL-based PCF-OM exhibits strong resilience to realistic fabrication deviations across all examined parameters. The optimal IL remains nearly unchanged, as indicated by the proximity of most yellow bars to the red reference line and the small associated IL differences. This high degree of fabrication tolerance is especially beneficial for practical photonic integration, which is particularly advantageous for real-world fabrication, where maintaining precise geometric accuracy can be difficult.

### 3.4. Comparative study between DQN-RL and traditional photonic design approaches

Table 7 provides a comprehensive comparison between the proposed modulator and representative state-of-the-art devices reported in the literature. Existing design and optimization approaches can be broadly classified into forward-designed structures optimized via manual parametric sweep studies (PSS) or experimental tuning^44,78–81^, and inverse-design strategies based on global or gradient-based optimization algorithms^82,83^. Forward PSS-based methods generally require extensive multidimensional parameter scanning and long device lengths, ranging from 2 μm to 500 μm, while often suffering from high IL or incomplete modulation performance. For instance, several PSS-optimized designs report IL values exceeding a few dB/mm^78,81^, or require device lengths on the order of several hundred micrometers^44^. Inverse-design techniques such as particle swarm optimization (PSO) and adjoint-based methods reduce reliance on manual tuning but still tend to result in relatively large device footprints (up to 362 μm). In particular, adjoint gradient-based optimization^1^is highly efficient for continuous parameter optimization due to its use of gradient information; however, it fundamentally relies on the differentiability of the design space and is less suitable for discrete or highly constrained geometrical parameters. Similarly, PSO^3^relies on population-based stochastic search, which may become less efficient as the design space grows. In contrast, the proposed DQN-RL framework operates effectively in a discrete and non-differentiable design space without requiring gradient information, while learning an optimization policy that balances exploration and exploitation. As a result, the proposed approach achieves a compact device length of 100 μm with an ultra-low IL of 0.935 dB/mm, which is among the lowest reported values in the compared works. Moreover, the globally optimized design is obtained within approximately 4 h for 1000 iterations, while the optimal solution is reached in approximately 12 min, whereas manual PSS optimization reported in^44^ requires extensive expert-driven iterative tuning over significantly longer time scales. These results demonstrate that the proposed DQN-RL framework provides an efficient and scalable approach for automated photonic modulator design.

**Table 7:** Comparative analysis of insertion IL and optimization time across various photonic modulator optimization methods.

Reference	Design	Method	LD (µm)	IL (dB/mm)	ER (dB/mm)	MD (%)	Time spent
Zhu, et al.^82^	inverse	PSO	N/A	0.93	N/A	N/A	N/A
AlAloul, et al.^78^	forward	PSS	12	516.67	~ 269	52.6	N/A
Zhang, et al.^83^	inverse	adjoint	362	31	N/A	N/A	N/A
Lee, et al.^84^	forward	Experimental	10.28	4.6	27.6	N/A	N/A
Fu, et al.^79^	forward	PSS	205	N/A	7	N/A	N/A
Joushaghani, et al.^80^	forward	Experimental	7	6	16.4	N/A	N/A
Younis, et al.^81^	forward	PSS	2	4.2	56	2.55	N/A
Dawood, et al.^44^	forward	PSS	500	2.6	~ 236	N/A	N/A
This work	inverse	DQN-RL	100	0.935	~ 280	99.9	4 h

## Conclusion

In this work, a DQN-based RL framework has been developed for the inverse design of a VO₂-integrated PCF-OM. The proposed approach eliminates the need for pre-collected datasets by directly interacting with the FDTD simulation environment and learning an optimization policy through experience. This significantly reduces the upfront computational cost associated with data generation and enables direct target-driven optimization within a discrete design space. The optimal solution is identified at an early stage, around 12 iterations (≈ 3 min), this behavior is consistent with the relatively small and discrete nature of the design space (3^5^=243 configurations). This performance is substantially faster compared to PSO (42 iterations, ≈ 13 min), random search (104 iterations, ≈ 28 min), grid search (21 iterations, ≈ 6 min), and Bayesian optimization (103 iterations, ≈ 33 min). The optimized DQN-RL-based PCF-OM demonstrates excellent modulation performance, achieving an ER exceeding 280 dB/mm, a maximum MD of 99.9%, and maintaining high ER and MD over a broad wavelength range from 3 to 7 μm. This broadband response highlights the effectiveness of the optimized geometry in enabling strong light–matter interaction with the VO₂ layer. A comprehensive fabrication tolerance analysis further confirms the robustness of the proposed design, with the IL remaining consistently below 1 dB/mm under independent parameter variations of ± 5%, demonstrating strong resilience against realistic manufacturing imperfections. These results confirm that, DQN-RL provides superior convergence speed, stability, and computational efficiency, while offering strong potential for extension to more complex and higher-dimensional photonic design problems.

## Data Availability

All data will be available upon reasonable request.
